# Survival of patients treated with radiation therapy for anaplastic astrocytoma

**DOI:** 10.2478/raon-2014-0019

**Published:** 2014-11-05

**Authors:** Christopher A. Barker, Maria Chang, Kathryn Beal, Timothy A. Chan

**Affiliations:** Department of Radiation Oncology, Memorial Sloan-Kettering Cancer Center, New York, NY, USA

**Keywords:** anaplastic astrocytoma, radiation therapy, prognosis, Radiation Therapy Oncology Group recursive partitioning analysis (RTOG RPA), temozolomide (TMZ), chemoradiation therapy

## Abstract

**Background:**

Anaplastic astrocytoma (AA) represents 7% of primary brain tumors in adults. Patient-, tumor-, and treatment-related factors are thought to be predictive of survival. We retrospectively assessed the association of patient-, tumor-, and treatment-related factors with survival in AA treated with radiotherapy (RT) at our institution.

**Patients and methods.:**

Medical records of patients with AA treated with RT between 1987 and 2007 were reviewed. Patient-, tumor-, and treatment-related variables were recorded and used to assign patients to a Radiation Therapy Oncology Group recursive partitioning analysis (RTOG RPA) classification. First use of chemotherapy was recorded. Log-rank tests and Cox regression models were used to assess for an association of patient-, tumor- and treatment-related factors with survival.

**Results:**

One-hundred twenty-six patients were eligible for study. Median age, Karnofsky performance status, and duration of symptoms were 43 years, 90, and 8 weeks. Median radiation dose was 59.4 Gy; 61% of patients underwent tumor resection, and 17% and 41% of patients received temozolomide during and after RT. Median survival was 31 months, and 2-year survival was 58%. RTOG RPA class was associated with survival (p < 0.001), but use of temozolomide during or after RT was not (p > 0.05).

**Conclusions:**

In this retrospective study with inherent limitations, RTOG RPA classification was associated with survival. Further studies are necessary to confirm or refute this finding.

## Introduction

According to the most recent statistical report of the Central Brain Tumor Registry of the United States, anaplastic astrocytoma (AA, a World Health Organization grade III glioma) is the fourth most common neuroepithelial brain tumor, with an incidence rate of 0.41 per 100,000 person years. This tumor accounts for 7% of all primary brain tumors in adults, with a 2-year survival rate of 43%.^1^

The treatment of patients with AA typically consists of maximal safe resection, followed by external beam radiation therapy (RT). This treatment approach is supported by observational data that suggest that the survival of patients with grade III primary brain tumors is longer after resection (versus biopsy alone).^2^ Randomized controlled trials of patients with grade III and IV glioma suggest that RT is associated with longer survival.^3^,^4^

The survival of patients diagnosed with AA and treated with RT has been associated with patient-, tumor-, and treatment-related factors. The Radiation Therapy Oncology Group (RTOG) conducted the most comprehensive analysis of prognostic factors in the largest group of patients with malignant gliomas (including astrocytomas with anaplastic or atypical foci) enrolled on prospective clinical research protocols and subjected these variables to recursive partitioning analysis (RPA). Six distinct prognostic classes were identified, with 2-year survival rates ranging from 4% to 76%, based on patient age, performance and neurologic functional status, mental status, duration of symptoms, extent of surgery, and RT dose.^5^

Given the poor survival rates of patients with AA, chemotherapy is often recommended. However, this point is controversial.^6^ A landmark study of patients with glioblastoma (GB, a World Health Organization grade IV glioma) demonstrated an improvement in survival with the use of temozolomide (TMZ, an oral alkylating chemotherapy) during and after RT.^7^,^8^ TMZ and RT have been widely used in the routine treatment of GB and successful outcomes have been reported from retrospective analyses.^9^ Because AA often transforms to GB, some have speculated that a similar upfront treatment approach is warranted in AA. Moreover, studies have demonstrated favorable results when TMZ is used for recurrent AA.^10^ However, the effect of using TMZ during and after RT for AA has not been well studied.^11^

The goal of this study was to describe the outcome of patients with AA that underwent RT, including an analysis of patient, tumor, and treatment-related factors known to be prognostic in malignant gliomas. In addition, we explored the benefit of TMZ, given during and after RT, to assess for effect on outcome.

## Patients and methods

### Patients and treatment

This retrospective clinical study was conducted with permission from the institutional review board at our institution. Eligible patients were ≥ 18 years old at the time of histologic diagnosis between 1987 and 2007, and were treated with external beam RT. Patients were identified in electronic institutional databases. Diagnosis of AA was confirmed by a neuropathologist at our institution. Molecular testing for genetic and epigenetic aberrations was not routinely performed during the study time period. Patients with secondary AA, inadequate medical records for review, or who did not receive external beam RT were excluded from study. Age at histologic diagnosis, Karnofsky performance status (KPS), neurologic functional status (able to work or not), mental status (mini-mental status exam score of ≥ 27 or notation of normal mental status), and duration of symptoms prior to histologic diagnosis were recorded. Extent of surgery (biopsy only, or neurosurgeon-determined subtotal or gross total resection), total RT dose (in Gy), and first use of TMZ or other chemotherapy (during and/or after RT) were recorded. Grade ≥ 4 toxicity was assessed using the National Cancer Institute’s Common Terminology Criteria for Adverse Events, version 4.0 (CTCAE).

Patient-, tumor-, and treatment-related characteristics were used to assign patients to a RTOG RPA classification.^5^,^12^ The criteria used for assignment to RTOG RPA class are presented in Table 1.

### Statistical analysis

Overall survival (OS) was defined as duration of time from the start of RT to death or last follow-up. OS was estimated using Kaplan-Meier methods. Log-rank tests were performed to compare survival between patients that did or did not receive TMZ during RT. Direct Cox regression models (p value limits in and out = 0.05) were built to evaluate the association of RTOG RPA and TMZ use with OS. Three models were built.

Model 1 analyzed the association of survival in patients that received concurrent TMZ during RT (n = 21) vs no TMZ during RT (n = 105)
Model 2 analyzed the association of survival in patients who received concurrent TMZ during RT (n = 21) vs no chemotherapy (TMZ or other) during RT (n = 94)
Model 3 analyzed the association of survival in patients that received TMZ at any time (during or after RT, n = 52) vs no TMZ use (n = 74)

Because the intent of TMZ use after RT could not clearly be defined as adjuvant (i.e., in the absence of disease progression) or salvage (i.e., in the presence of disease progression) therapy, no distinction in the analysis was made for patients that may have received TMZ at time of progression. Hazard ratios (HRs) along with 95% confidence intervals (CIs) were reported. Analyses were carried out using WinSTAT^®^ for Microsoft® Excel (Version 2009.1).

## Results

One-hundred twenty-six patients met the criteria for study. Median follow-up was 28 months. Thirty-six patients were alive at time of last follow-up, and had been followed for a median of 72 months. Median age was 43 years (range, 19–79 years). Median KPS was 90 (range, 50–100). Median duration of symptoms prior to diagnosis was 8 weeks (range 0–312 weeks). Median radiation dose was 59.4 Gy (range, 16–120 Gy). Baseline patient and treatment-related characteristics are presented in Table 2.

**FIGURE 1.**
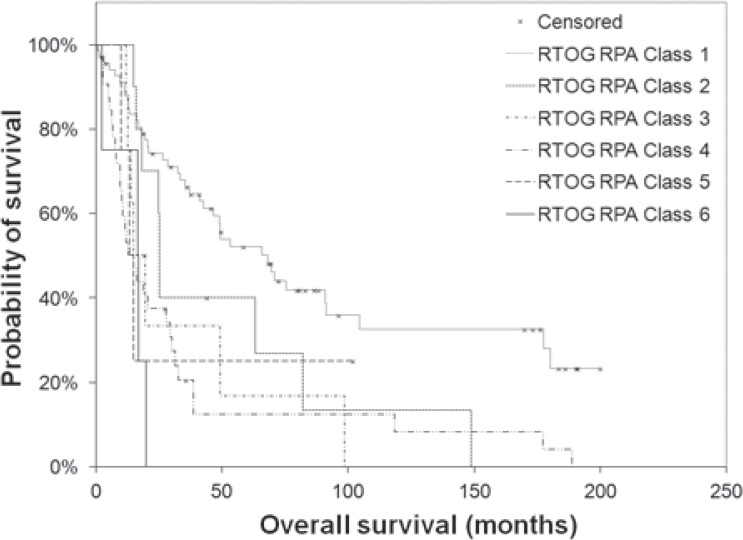
Survival of patients with anaplastic astrocytoma treated with radiation therapy, by Radiation Therapy Oncology Group recursive partitioning analysis (RTOG RPA) classification (n = 126). The log-rank test revealed a statistically significant difference in survival by RTOG RPA classification (p < 0.001).

Median OS duration was 31 months, and 2-year OS was 58%. Using the aforementioned patient-and treatment-related criteria, patients were assigned to a RTOG RPA class. The median duration of OS and 2-year OS rates by RTOG RPA class for the present cohort are displayed alongside reported data from the RTOG in Table 3. The log-rank test revealed a statistically significant difference in survival among the six classes in the present cohort (p < 0.001), as displayed in Figure 1.

**FIGURE 2.**
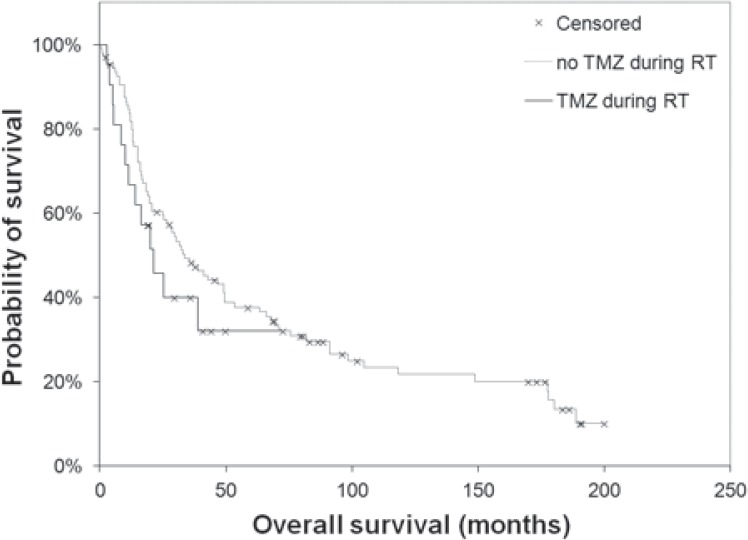
Survival of patients with anaplastic astrocytoma treated with radiation therapy, by concurrent use of temozolomide use during radiotherapy (n = 21) or no use of temozolomide during radiotherapy (n = 105). The log-rank test revealed no difference in survival by use or non-use of temozolomide during radiation therapy (RT; p = 0.28).

The log-rank test revealed no difference in survival between patients that were or were not taking TMZ during RT (p = 0.28), as displayed in Figure 2. Median survival of patients receiving TMZ during RT was 19 months, and median survival of patients not receiving TMZ during RT was 33 months; 2-year survival of patients receiving TMZ during was 46%, and 2-year survival of patients not receiving TMZ during RT was 60%.

Cox regression model 1 revealed an association of survival with RTOG RPA class (HR, 1.40; 95% CI, 1.27–1.53; p < 0.001), but not use of concurrent TMZ during RT (HR, 1.40; 95% CI, 0.80–2.00; p = 0.27). Cox regression model 2 demonstrated an association of survival with RTOG RPA class (HR, 1.35; 95% CI, 1.22–1.49; p < 0.001), but not use of concurrent TMZ during RT (HR, 1.34; 95% CI, 0.74–1.95; p = 0.34). Similarly, Cox regression model 3 revealed an association of survival with RTOG RPA class (HR, 1.41; 95% CI, 1.28–1.54; p < 0.001), but not use of TMZ at any time (HR, 1.09; 95% CI, 0.65–1.54; p = 0.70).

Mild-moderate toxicity (CTCAE grade 1–2) was common and consisted of fatigue, alopecia, headaches, nausea, vomiting, cognitive impairment, and disturbances. One patient developed acute lymphoblastic leukemia 4 years after receiving RT followed by carmustine chemotherapy. She died of infectious neutropenia during therapy for acute lymphoblastic leukemia.

## Discussion

In this study, we sought to characterize the outcome of patients with AA treated with RT at our institution. We found that previously reported patient-, tumor-, and treatment-related factors prognostic of survival in patients enrolled on large clinical trials were prognostic in the present cohort. We also attempted to determine the effect of TMZ chemotherapy on the outcome of patients treated for AA. We did not find an association of TMZ with improved survival.

The RTOG RPA classification system, reported by Curran *et al*. in 1993, is a widely used system for assessing prognosis in patients with malignant glioma, being cited over 600 times in the medical literature. Using 20 patient-related, 3 tumor-related, and 6 treatment-related variables, the authors performed an RPA on a group of 1578 patients with malignant glioma, and created a regression tree of prognostic variables that classified patients into six homogenous subsets by survival. Eighteen percent of patients in that analysis harbored an astrocytoma with anaplastic or atypical foci.^5^ While the RTOG RPA was validated in another cohort of patients with malignant glioma, to our knowledge the present report is the first describing validation in a retrospective cohort of patient with AA only.^12^ The distribution of patients in the present cohort includes more patients with favorable prognoses. However, median and 2-year OS rates were relatively similar except in the poor-prognosis categories (RTOG RPA classes 5 and 6), where the present cohort demonstrated superior survival (albeit in a very small number of patients). In the present study, RTOG RPA class assignment was able to predict a statistically significant difference in survival between the groups. Determining prognosis based on patient-, tumor-, and treatment-related variables is helpful when trying to determine if newer therapies are associated with differences in survival.

Because of the landmark study demonstrating that TMZ use during and after RT improves survival in patients with GB, several studies employing a similar treatment paradigm have been conducted in patients with AA.^8^ Kim *et al*. described 33 patients with grade III gliomas treated with TMZ during and after RT. 11 Sixty-five percent of patients in the study were treated for AA. The authors demonstrated the regimen to be safe and well tolerated, with grade ≥3 hematologic toxicity occurring in 15% of patients treated with TMZ during RT, and in 9% of patients treated with TMZ after RT. A specific analysis of the outcomes of patients with AA was not performed.

Combs *et al*. performed a retrospective matched-pair analysis of the outcomes of 60 patients with anaplastic astrocytic tumors treated with RT. Twenty patients who received TMZ during RT were matched to 40 historical controls treated with RT alone. Matching was done based on patient age (<50 years, or ≥50 years), extent of resection (complete, subtotal, or biopsy), and histologic subtype (pure AA, anaplastic oligoastrocytoma, and anaplastic oligodendroglioma). The majority of the patients studied had AA (90%). Median age was 42.4–44.5 years (range, 7–77), with all patients having KPS ≥70, and 45% having biopsy without tumor resection, thereby representing a cohort very similar to the present study. The authors found median survival of their cohort to be 14 months from time of histologic diagnosis, with 2-year survival of 36%. In their study, extent of surgical re-section was not associated with longer survival. The use of TMZ during RT was not associated with longer overall or progression-free survival.^13^ The present study corroborates these results, finding no association of TMZ use during or after RT with an improvement in survival.

The present study is limited by several factors. First, the present cohort is relatively small, which limits the power of the analysis. This factor is inherent in retrospective clinical research of a rare disease like AA. It should be noted that this series represents the largest single-institution cohort of AA patients in the contemporary era treated with TMZ. We cannot exclude that bias in the selection of therapy may have led to the observed associations of treatment and outcome in this study. For example, TMZ may have been selected as part of a more aggressive therapeutic regimen for patients with an anticipated worse outcome. By incorporating RTOG RPA into the multivariable analysis, we attempted to minimize this confounding factor. Moreover, additional features prognostic of survival (independent of therapy) were not routinely assessed. Studies have demonstrated that radiographic features (tumor location, size, and ring enhancement), histopathologic features (proliferation rate), and biologic markers (O-6-methylguanine methyltransferase methylation) are also likely to be prognostic of outcome and predictive of response to therapy in this disease.^14^–^16^

The efficacy of TMZ chemotherapy has been demonstrated in patients with recurrent AA.^10^ Many practitioners recommend TMZ during or after RT for AA based on extrapolation from trials in GB with the hope of optimizing the outcome of patients with an otherwise poor long-term prognosis. However, the benefit of using TMZ during or after RT as adjuvant therapy has not been clearly demonstrated. Other studies have suggested that more intensive therapy is not beneficial in patients with AA.^17^–^18^ While potentially controversial, the present findings suggest that TMZ may be best reserved for use in the setting of AA recurrence. The ongoing European Organisation for Research and Treatment of Cancer 26053-22054 CATNON intergroup trial (NCT00626990) will help clarify the appropriate use of TMZ in patients with AA. This 4-arm, multicenter, randomized trial will assess the benefit of TMZ given concurrently with RT, after RT, or both during and after RT in patients with anaplastic gliomas without chromosome 1p/19q deletion. Until further well-controlled studies of this type are reported, the recommendation for TMZ in addition to RT deserves careful discussion between patients and their physicians.

## Figures and Tables

**TABLE 1.** Criteria for classification of patients with anaplastic astrocytoma to a Radiation Therapy Oncology Group recursive partitioning analysis (RTOG RPA) classification

**RTOG RPA Classification**	**Criteria for assignment to classification**			

	**Age**	**Mental status**	**KPS**	**Duration of symptoms prior to diagnosis**
1	< 50 years	Normal		
2	≥ 50 years		≥ 70	> 3 months
3	< 50 years	Abnormal		
4	≥ 50 years		≥ 70	≥ 3 months
5	≥ 50 years	Normal	< 70	
6	≥ 50 years	Abnormal	< 70	

RTOG RPA = Radiation Therapy Oncology Group recursive partitioning analysis; KPS = Karnofsky performance status.

**TABLE 2.** Baseline patient and treatment-related characteristics of the patients studied (n = 126)

**Patient characteristics**		**N**	**%**

Age (years)	19–30	29	23%
	31–40	25	20%
	41–50	24	19%
	51–60	19	15%
	61–70	19	15%
	71–79	10	8%
KPS	100	9	7%
	90	60	48%
	80	36	29%
	70	11	9%
	60	9	7%
	50	1	1%
Mental status	Normal	101	80%
	Abnormal	25	20%
Symptom duration before diagnosis (weeks)	0–4	48	38%
	5–12	37	29%
	> 12	40	32%
	Unknown	1	1%
Able to work	Yes	44	35%
	No	80	63%
	Unknown	2	2%

**Treatment characteristics**		**N**	**%**

Extent of surgery	Biopsy	49	39%
	Subtotal resection	50	40%
	Gross total resection	27	21%
RT dose (Gy)	≥ 72	4	3%
	55.8–60.2	110	87%
	≥ 50.4	12	10%
Chemotherapy during RT	None	94	75%
	Temozolomide	21	17%
	Other	11	9%
First chemotherapy after RT	Temozolomide	52	41%
	Other	55	44%
	None	13	10%
	Unknown	6	5%

KPS = Karnofsky performance status; RT = radiation therapy

**TABLE 3.** Distribution, median and 2-year overall survival of patients by Radiation Therapy Oncology Group (RTOG RPA) classification in the present study, and compared to historical controls from the RTOG database

**Present study**					**Historical comparison**					

					**RTOG database (RTOG 74-01, 79-18, 83-02)**					
**RTOG RPA Class**	**N**	**%**	**Median OS (months)**	**2-year OS (%)**	**N**	**%**	**Median OS (months)**	**95% CI**	**2-year OS (%)**	**95% CI**
1	68	54%	66	73	139	10%	58.6	46.8–108.1	76	68.7–83.3
2	10	8%	25	70	34	2%	37.4	26.2–45.9	68	51.6–83.6
3	8	6%	15	33	175	12%	17.9	15.5–20.6	35	18.6–42.0
4	32	25%	13	37	457	31%	11.1	10.4–11.9	15	12.0–18.0
5	4	3%	13	25	395	27%	8.9	8.3–9.5	6	4.0–8.0
6	4	3%	17	0	263	18%	4.6	4.3–5.3	4	1.8–6.2

RTOG = Radiation Therapy Oncology Group; RPA = recursive partitioning analysis; OS = overall survival; 95% CI = 95% confidence interval
